# Dichlorido(*N*,*N*′-diisopropyl­piperidine-1-carboximidamidato-κ^2^ *N*,*N*′)titanium(IV)

**DOI:** 10.1107/S1600536810053262

**Published:** 2010-12-24

**Authors:** Mei Wang, Ying-Ming Yao, Yong Zhang, Zhen-Qin Zhang, Li-Ying Shi

**Affiliations:** a Jiangpu Senior Middle School, Nanjing 211800, Jiangsu Province, People’s Republic of China; b Key Laboratory of Organic Synthesis of Jiangsu Province, Department of Chemistry and Chemical Engineering, Dushu Lake Campus, Suzhou University, Suzhou 215123, Jiangsu Province, People’s Republic of China; c School of Pharmacy, Nanjing Medical University, Nanjing 210029, Jiangsu Province, People’s Republic of China

## Abstract

In the mononuclear title complex, [Ti(C_12_H_24_N_3_)_2_Cl_2_], the Ti^IV^ ion, located on a crystallographic inversion center, is six-coordinated by four N atoms from two *N*′,*N*′′-diisopropyl-*N*-carboxamidine anions and two chloride atoms in a distorted octahedral geometry. The dihedral angles between the piperidine groups and the NCN chelate rings are 51.5 (1) and 52.3 (1)°.

## Related literature

For background to the coordination chemistry of guanidinates, see: Braunschweig *et al.* (2010 ▶). For the synthesis of similar compounds, see: Bailey *et al.* (2000 ▶); Mullins *et al.* (2001 ▶). 
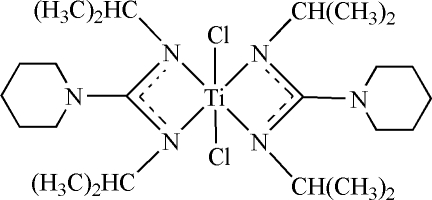


## Experimental

### 

#### Crystal data

[Ti(C_12_H_24_N_3_)_2_Cl_2_]
*M* *_r_* = 539.48
Triclinic, 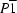

*a* = 8.2810 (3) Å
*b* = 13.3678 (9) Å
*c* = 13.6178 (7) Å
α = 86.266 (10)°
β = 75.841 (8)°
γ = 82.304 (8)°
*V* = 1447.69 (13) Å^3^
*Z* = 2
Mo *K*α radiation
μ = 0.50 mm^−1^
*T* = 193 K
0.70 × 0.25 × 0.15 mm

#### Data collection

Rigaku Mercury diffractometer
Absorption correction: multi-scan (*REQAB*; Jacobson, 1998 ▶) *T*_min_ = 0.720, *T*_max_ = 0.928
14490 measured reflections
6513 independent reflections
5896 reflections with *I* > 2σ(*I*)
*R*_int_ = 0.024

#### Refinement

*R*[*F* ^2^ > 2σ(*F* ^2^)] = 0.041
*wR*(*F* ^2^) = 0.097
*S* = 1.08
6513 reflections
306 parameters
H-atom parameters constrained
Δρ_max_ = 0.64 e Å^−3^
Δρ_min_ = −0.32 e Å^−3^

### 

Data collection: *CrystalClear* (Rigaku, 1999 ▶); cell refinement: *CrystalClear*; data reduction: *CrystalStructure* (Rigaku/MSC, 2002 ▶); program(s) used to solve structure: *SHELXS97* (Sheldrick, 2008 ▶); program(s) used to refine structure: *SHELXL97* (Sheldrick, 2008 ▶); molecular graphics: *ORTEPIII* (Burnett & Johnson, 1996 ▶); software used to prepare material for publication: *CrystalStructure*.

## Supplementary Material

Crystal structure: contains datablocks I, global. DOI: 10.1107/S1600536810053262/hg2762sup1.cif

Structure factors: contains datablocks I. DOI: 10.1107/S1600536810053262/hg2762Isup2.hkl

Additional supplementary materials:  crystallographic information; 3D view; checkCIF report

## Figures and Tables

**Fig. 1.**
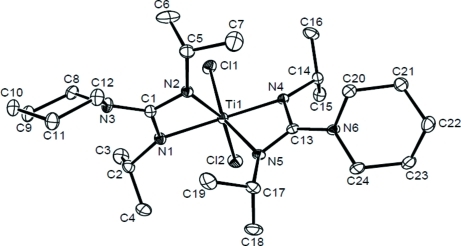
Crystal structure and atom numbering of the title compound, shown with 30% probability displacement ellipsoids.

## supplementary crystallographic information

### Comment

Guanidinate anions, [(RN)_2_C(NR'_2_)]^-^, are isoelectronic alternatives to cyclopentadienyl ligands and modifications to their electronic properties and steric bulk can be investigated through variation of the substiuents on the nitrogen atoms. As a result, guanidinate ligands have been attacted increasing attention as ancillary ligands in the coordination and organometallic chemistry of main group and transition metals (Braunschweig *et al.*, 2010). As part of our ongoing investigations in this field we report here the crystal structure of the title compound. In the crystal structure of the title compound the Ti atom is coordinated by four nitrogen atoms of two guanidinate ligands and two chloride atoms within a triclinic coordination symmetry (Figure 1).

The Ti—N bond lengths vary from 2.0125 (14) to 2.1299 (14)Å, which are close to the values reported for [Et_2_NC(NPh)_2_]_2_TiCl_2_ (Bailey *et al.*, 2000) and [Et_2_NC(N^i^Pr)_2_]_2_TiCl_2_ and [Et_2_NC(N^i^Pr)_2_]_2_TiS_2_ (Mullins *et al.*, 2001). The bond lengths of Ti—Cl of 2.3254 (5) and 2.3308 (6)Å are comparable with those in [Et_2_NC(NPh)_2_]_2_TiCl_2_ and [Et_2_NC(N^i^Pr)_2_]_2_TiCl_2_. The bond distance around N(1), C(1), N(2) and N(4), C(13), N(5) average 1.342Å indicating partial double-bonding character and a π-conjugated NCN chelate. The bond angles around N(3) and N(6) ranging from 113.9 (1) to 124.5 (1)° consistent with *sp*^2^-hybridized nitrogen atoms. The dihedral angles between the piperidine groups and the NCN chelate rings are 51.5 and 52.3°.

### Experimental

A solution of n-butyllithium (8.40 ml, 10 mmol) in hexane was added *via* syringe to a THF solution of piperidine (10 mmol) at -78°C. The solution was warmed to room temperature slowly and stirred for 30 min. Then *N*-((isopropylimino)methylene)propan-2-amine (1.26 g, 10 mmol) was added *via* syringe at 0°C. The mixture was stirred at room temperature (r.t.) for 30 min, then was added to a suspension of TiCl_4_(THF)_2 _(1.65 g, 5 mmol) in toluene (20 ml) at r.t. The reaction mixture was stirred overnight at r.t. After removal of volatiles under vacuum, the dark red residue was extracted with toluene, and LiCl was removed by centrifugation. The dark red crystals were obtained from concentrated toluene solution at -10°C. Anal. Calcd. for C_24_H_48_Cl_2_N_6_Ti: C, 53.50; H, 8.91; N, 15.59. Found: C, 53.81; H, 9.58; N, 15.86%.

### Refinement

H atoms of the methyl groups were placed geometrically with C—H = 0.97 Å and allowed to ride during subsequent refinement with *U*_iso_(H) = 1.5U_eq_(C). Fifteen missing reflections appeared to be obscured by the beamstop.

### Crystal data

**Table:**

[Ti(C_12_H_24_N_3_)_2_Cl_2_]	*Z* = 2
*M**_r_* = 539.48	*F*(000) = 580
Triclinic, *P*1	*D*_x_ = 1.238 Mg m^−^^3^
Hall symbol: -P 1	Mo *K*α radiation, λ = 0.71070 Å
*a* = 8.2810 (3) Å	Cell parameters from 3913 reflections
*b* = 13.3678 (9) Å	θ = 3.1–27.5°
*c* = 13.6178 (7) Å	µ = 0.50 mm^−^^1^
α = 86.266 (10)°	*T* = 193 K
β = 75.841 (8)°	Block, dark-red
γ = 82.304 (8)°	0.70 × 0.25 × 0.15 mm
*V* = 1447.69 (13) Å^3^	

### Data collection

**Table:**

Rigaku Mercury diffractometer	6513 independent reflections
Radiation source: fine-focus sealed tube	5896 reflections with *I* > 2σ(*I*)
graphite	*R*_int_ = 0.024
Detector resolution: 14.62 pixels mm^-1^	θ_max_ = 27.5°, θ_min_ = 3.1°
ω scans	*h* = −10→10
Absorption correction: multi-scan (REQAB; Jacobson, 1998)	*k* = −17→17
*T*_min_ = 0.720, *T*_max_ = 0.928	*l* = −16→17
14490 measured reflections	

### Refinement

**Table:**

Refinement on *F*^2^	Primary atom site location: structure-invariant direct methods
Least-squares matrix: full	Secondary atom site location: difference Fourier map
*R*[*F*^2^ > 2σ(*F*^2^)] = 0.041	Hydrogen site location: inferred from neighbouring sites
*wR*(*F*^2^) = 0.097	H-atom parameters constrained
*S* = 1.08	*w* = 1/[σ^2^(*F*_o_^2^) + (0.0406*P*)^2^ + 0.8209*P*] where *P* = (*F*_o_^2^ + 2*F*_c_^2^)/3
6513 reflections	(Δ/σ)_max_ < 0.001
306 parameters	Δρ_max_ = 0.64 e Å^−^^3^
0 restraints	Δρ_min_ = −0.32 e Å^−^^3^

### Special details

**Table:**

Geometry. All e.s.d.'s (except the e.s.d. in the dihedral angle between two l.s. planes) are estimated using the full covariance matrix. The cell e.s.d.'s are taken into account individually in the estimation of e.s.d.'s in distances, angles and torsion angles; correlations between e.s.d.'s in cell parameters are only used when they are defined by crystal symmetry. An approximate (isotropic) treatment of cell e.s.d.'s is used for estimating e.s.d.'s involving l.s. planes.
Refinement. Refinement of *F*^2^ against ALL reflections. The weighted *R*-factor *wR* and goodness of fit *S* are based on *F*^2^, conventional *R*-factors *R* are based on *F*, with *F* set to zero for negative *F*^2^. The threshold expression of *F*^2^ > σ(*F*^2^) is used only for calculating *R*-factors(gt) *etc*. and is not relevant to the choice of reflections for refinement. *R*-factors based on *F*^2^ are statistically about twice as large as those based on *F*, and *R*- factors based on ALL data will be even larger.

### Fractional atomic coordinates and isotropic or equivalent isotropic displacement parameters (Å^2^)

**Table:**

	*x*	*y*	*z*	*U*_iso_*/*U*_eq_	
Ti1	0.02434 (4)	0.22060 (2)	0.23396 (2)	0.02022 (8)	
Cl1	−0.06242 (6)	0.23150 (3)	0.08319 (4)	0.03338 (12)	
Cl2	−0.22892 (6)	0.17162 (4)	0.32905 (4)	0.03487 (12)	
N1	−0.04278 (17)	0.36915 (10)	0.27568 (11)	0.0234 (3)	
N2	0.20565 (17)	0.31810 (10)	0.17994 (11)	0.0227 (3)	
N3	0.12949 (17)	0.49848 (10)	0.20127 (12)	0.0255 (3)	
N4	0.16572 (17)	0.08674 (10)	0.20244 (10)	0.0194 (3)	
N5	0.15650 (17)	0.15914 (10)	0.34437 (10)	0.0216 (3)	
N6	0.34501 (17)	0.00317 (11)	0.30397 (10)	0.0223 (3)	
C1	0.0996 (2)	0.39864 (12)	0.21722 (13)	0.0218 (3)	
C2	−0.1776 (2)	0.43961 (13)	0.33551 (14)	0.0278 (4)	
H2	−0.1393	0.5081	0.3280	0.033*	
C3	−0.3349 (2)	0.44610 (17)	0.29507 (18)	0.0405 (5)	
H3A	−0.3080	0.4662	0.2230	0.061*	
H3B	−0.4214	0.4962	0.3326	0.061*	
H3C	−0.3764	0.3800	0.3036	0.061*	
C4	−0.2153 (3)	0.40813 (17)	0.44766 (15)	0.0408 (5)	
H4A	−0.2479	0.3397	0.4563	0.061*	
H4B	−0.3073	0.4551	0.4853	0.061*	
H4C	−0.1150	0.4094	0.4735	0.061*	
C5	0.3538 (2)	0.32465 (15)	0.09379 (15)	0.0329 (4)	
H5	0.4166	0.3781	0.1095	0.039*	
C6	0.3042 (3)	0.3572 (2)	−0.00399 (17)	0.0558 (7)	
H6A	0.2423	0.3064	−0.0220	0.084*	
H6B	0.4052	0.3641	−0.0580	0.084*	
H6C	0.2328	0.4222	0.0045	0.084*	
C7	0.4687 (3)	0.2273 (2)	0.0867 (2)	0.0675 (9)	
H7A	0.4964	0.2101	0.1522	0.101*	
H7B	0.5718	0.2347	0.0346	0.101*	
H7C	0.4129	0.1734	0.0687	0.101*	
C8	0.0227 (2)	0.57243 (13)	0.15365 (14)	0.0288 (4)	
H8A	−0.0868	0.5475	0.1600	0.035*	
H8B	0.0762	0.5804	0.0806	0.035*	
C9	−0.0054 (2)	0.67433 (14)	0.20283 (17)	0.0350 (4)	
H9A	−0.0738	0.6687	0.2731	0.042*	
H9B	−0.0678	0.7247	0.1648	0.042*	
C10	0.1618 (3)	0.70969 (14)	0.20385 (16)	0.0351 (4)	
H10A	0.2226	0.7255	0.1336	0.042*	
H10B	0.1411	0.7721	0.2427	0.042*	
C11	0.2692 (3)	0.62867 (14)	0.25152 (16)	0.0349 (4)	
H11A	0.2144	0.6185	0.3241	0.042*	
H11B	0.3799	0.6512	0.2471	0.042*	
C12	0.2936 (2)	0.52900 (14)	0.19807 (16)	0.0319 (4)	
H12A	0.3567	0.5372	0.1268	0.038*	
H12B	0.3589	0.4764	0.2321	0.038*	
C13	0.22731 (19)	0.08123 (12)	0.28716 (12)	0.0192 (3)	
C14	0.1624 (2)	−0.00526 (12)	0.14861 (13)	0.0234 (3)	
H14	0.2350	−0.0617	0.1743	0.028*	
C15	−0.0156 (2)	−0.03373 (14)	0.17258 (15)	0.0310 (4)	
H15A	−0.0539	−0.0470	0.2457	0.046*	
H15B	−0.0170	−0.0945	0.1364	0.046*	
H15C	−0.0904	0.0220	0.1511	0.046*	
C16	0.2324 (3)	0.00808 (16)	0.03502 (14)	0.0353 (4)	
H16A	0.1663	0.0651	0.0086	0.053*	
H16B	0.2267	−0.0536	0.0015	0.053*	
H16C	0.3495	0.0213	0.0218	0.053*	
C17	0.2158 (2)	0.18903 (14)	0.43013 (13)	0.0281 (4)	
H17	0.3158	0.1406	0.4370	0.034*	
C18	0.0777 (3)	0.18062 (18)	0.52705 (14)	0.0398 (5)	
H18A	−0.0252	0.2224	0.5191	0.060*	
H18B	0.1135	0.2040	0.5842	0.060*	
H18C	0.0563	0.1100	0.5400	0.060*	
C19	0.2684 (3)	0.29504 (16)	0.41280 (17)	0.0398 (5)	
H19A	0.3570	0.2976	0.3503	0.060*	
H19B	0.3105	0.3119	0.4702	0.060*	
H19C	0.1714	0.3437	0.4067	0.060*	
C20	0.4988 (2)	−0.01999 (13)	0.22347 (13)	0.0265 (4)	
H20A	0.5903	0.0133	0.2375	0.032*	
H20B	0.4786	0.0075	0.1577	0.032*	
C21	0.5530 (2)	−0.13335 (14)	0.21646 (14)	0.0315 (4)	
H21A	0.4696	−0.1656	0.1924	0.038*	
H21B	0.6625	−0.1459	0.1669	0.038*	
C22	0.5683 (2)	−0.17964 (16)	0.31945 (16)	0.0366 (4)	
H22A	0.6621	−0.1541	0.3394	0.044*	
H22B	0.5932	−0.2540	0.3152	0.044*	
C23	0.4055 (3)	−0.15274 (15)	0.39905 (15)	0.0369 (4)	
H23A	0.4191	−0.1805	0.4662	0.044*	
H23B	0.3141	−0.1839	0.3823	0.044*	
C24	0.3581 (3)	−0.03855 (15)	0.40407 (13)	0.0330 (4)	
H24A	0.2495	−0.0230	0.4537	0.040*	
H24B	0.4445	−0.0076	0.4266	0.040*	

### Atomic displacement parameters (Å^2^)

**Table:**

	*U*^11^	*U*^22^	*U*^33^	*U*^12^	*U*^13^	*U*^23^
Ti1	0.01973 (15)	0.01908 (15)	0.02277 (16)	−0.00208 (11)	−0.00691 (11)	−0.00083 (11)
Cl1	0.0400 (3)	0.0334 (2)	0.0332 (2)	−0.00592 (19)	−0.0216 (2)	0.00408 (18)
Cl2	0.0250 (2)	0.0379 (2)	0.0392 (3)	−0.00926 (18)	0.00079 (18)	−0.0037 (2)
N1	0.0179 (6)	0.0208 (7)	0.0295 (8)	−0.0010 (5)	−0.0023 (6)	−0.0025 (6)
N2	0.0198 (7)	0.0215 (7)	0.0248 (7)	−0.0015 (5)	−0.0023 (5)	−0.0006 (5)
N3	0.0210 (7)	0.0194 (7)	0.0380 (8)	−0.0030 (5)	−0.0114 (6)	0.0032 (6)
N4	0.0210 (6)	0.0203 (6)	0.0184 (6)	−0.0015 (5)	−0.0082 (5)	−0.0010 (5)
N5	0.0222 (7)	0.0257 (7)	0.0186 (7)	−0.0027 (5)	−0.0075 (5)	−0.0033 (5)
N6	0.0213 (7)	0.0280 (7)	0.0172 (7)	0.0015 (5)	−0.0065 (5)	0.0011 (5)
C1	0.0211 (8)	0.0212 (8)	0.0248 (8)	−0.0020 (6)	−0.0090 (6)	−0.0007 (6)
C2	0.0222 (8)	0.0250 (8)	0.0327 (9)	0.0023 (7)	−0.0017 (7)	−0.0043 (7)
C3	0.0208 (9)	0.0439 (12)	0.0536 (13)	0.0020 (8)	−0.0062 (9)	−0.0008 (10)
C4	0.0407 (11)	0.0410 (11)	0.0324 (11)	0.0045 (9)	0.0030 (9)	−0.0046 (9)
C5	0.0255 (9)	0.0319 (9)	0.0355 (10)	−0.0036 (7)	0.0033 (8)	−0.0003 (8)
C6	0.0425 (13)	0.088 (2)	0.0304 (11)	−0.0109 (13)	0.0037 (10)	0.0027 (12)
C7	0.0363 (13)	0.0521 (15)	0.087 (2)	0.0078 (11)	0.0249 (13)	0.0141 (14)
C8	0.0292 (9)	0.0262 (9)	0.0326 (9)	−0.0020 (7)	−0.0129 (8)	0.0056 (7)
C9	0.0336 (10)	0.0228 (9)	0.0473 (12)	0.0004 (7)	−0.0105 (9)	0.0038 (8)
C10	0.0402 (11)	0.0210 (8)	0.0439 (11)	−0.0056 (8)	−0.0091 (9)	0.0010 (8)
C11	0.0359 (10)	0.0274 (9)	0.0461 (12)	−0.0084 (8)	−0.0164 (9)	−0.0005 (8)
C12	0.0243 (9)	0.0257 (9)	0.0475 (11)	−0.0062 (7)	−0.0112 (8)	0.0015 (8)
C13	0.0177 (7)	0.0228 (8)	0.0180 (7)	−0.0052 (6)	−0.0051 (6)	0.0018 (6)
C14	0.0262 (8)	0.0208 (8)	0.0260 (8)	0.0000 (6)	−0.0123 (7)	−0.0036 (6)
C15	0.0331 (9)	0.0257 (9)	0.0399 (11)	−0.0074 (7)	−0.0176 (8)	−0.0008 (7)
C16	0.0387 (10)	0.0429 (11)	0.0251 (9)	0.0011 (9)	−0.0094 (8)	−0.0110 (8)
C17	0.0279 (9)	0.0350 (9)	0.0250 (9)	0.0000 (7)	−0.0132 (7)	−0.0086 (7)
C18	0.0432 (11)	0.0547 (13)	0.0212 (9)	0.0020 (10)	−0.0097 (8)	−0.0078 (9)
C19	0.0419 (11)	0.0403 (11)	0.0456 (12)	−0.0074 (9)	−0.0219 (10)	−0.0131 (9)
C20	0.0200 (8)	0.0308 (9)	0.0278 (9)	−0.0030 (7)	−0.0054 (7)	0.0034 (7)
C21	0.0239 (9)	0.0341 (10)	0.0328 (10)	0.0037 (7)	−0.0036 (7)	−0.0002 (8)
C22	0.0316 (10)	0.0347 (10)	0.0418 (11)	0.0062 (8)	−0.0127 (9)	0.0052 (8)
C23	0.0423 (11)	0.0366 (10)	0.0279 (10)	0.0036 (9)	−0.0079 (8)	0.0089 (8)
C24	0.0405 (10)	0.0382 (10)	0.0203 (9)	0.0033 (8)	−0.0127 (8)	0.0028 (7)

### Geometric parameters (Å, °)

**Table:**

Ti1—N4	2.0125 (14)	C9—C10	1.525 (3)
Ti1—N1	2.0669 (14)	C9—H9A	0.9900
Ti1—N2	2.0864 (14)	C9—H9B	0.9900
Ti1—N5	2.1299 (14)	C10—C11	1.522 (3)
Ti1—Cl1	2.3254 (5)	C10—H10A	0.9900
Ti1—Cl2	2.3308 (6)	C10—H10B	0.9900
Ti1—C1	2.5224 (17)	C11—C12	1.526 (3)
Ti1—C13	2.5303 (16)	C11—H11A	0.9900
N1—C1	1.343 (2)	C11—H11B	0.9900
N1—C2	1.473 (2)	C12—H12A	0.9900
N2—C1	1.337 (2)	C12—H12B	0.9900
N2—C5	1.483 (2)	C14—C16	1.521 (2)
N3—C1	1.383 (2)	C14—C15	1.524 (2)
N3—C12	1.461 (2)	C14—H14	1.0000
N3—C8	1.464 (2)	C15—H15A	0.9800
N4—C13	1.365 (2)	C15—H15B	0.9800
N4—C14	1.479 (2)	C15—H15C	0.9800
N5—C13	1.324 (2)	C16—H16A	0.9800
N5—C17	1.469 (2)	C16—H16B	0.9800
N6—C13	1.377 (2)	C16—H16C	0.9800
N6—C24	1.462 (2)	C17—C19	1.525 (3)
N6—C20	1.475 (2)	C17—C18	1.529 (3)
C2—C3	1.523 (3)	C17—H17	1.0000
C2—C4	1.525 (3)	C18—H18A	0.9800
C2—H2	1.0000	C18—H18B	0.9800
C3—H3A	0.9800	C18—H18C	0.9800
C3—H3B	0.9800	C19—H19A	0.9800
C3—H3C	0.9800	C19—H19B	0.9800
C4—H4A	0.9800	C19—H19C	0.9800
C4—H4B	0.9800	C20—C21	1.524 (3)
C4—H4C	0.9800	C20—H20A	0.9900
C5—C7	1.500 (3)	C20—H20B	0.9900
C5—C6	1.508 (3)	C21—C22	1.523 (3)
C5—H5	1.0000	C21—H21A	0.9900
C6—H6A	0.9800	C21—H21B	0.9900
C6—H6B	0.9800	C22—C23	1.526 (3)
C6—H6C	0.9800	C22—H22A	0.9900
C7—H7A	0.9800	C22—H22B	0.9900
C7—H7B	0.9800	C23—C24	1.526 (3)
C7—H7C	0.9800	C23—H23A	0.9900
C8—C9	1.522 (3)	C23—H23B	0.9900
C8—H8A	0.9900	C24—H24A	0.9900
C8—H8B	0.9900	C24—H24B	0.9900
			
N4—Ti1—N1	159.82 (6)	C8—C9—H9A	109.5
N4—Ti1—N2	100.06 (6)	C10—C9—H9A	109.5
N1—Ti1—N2	63.86 (5)	C8—C9—H9B	109.5
N4—Ti1—N5	64.01 (5)	C10—C9—H9B	109.5
N1—Ti1—N5	102.08 (6)	H9A—C9—H9B	108.1
N2—Ti1—N5	89.81 (5)	C11—C10—C9	110.83 (15)
N4—Ti1—Cl1	93.71 (4)	C11—C10—H10A	109.5
N1—Ti1—Cl1	99.11 (4)	C9—C10—H10A	109.5
N2—Ti1—Cl1	93.32 (4)	C11—C10—H10B	109.5
N5—Ti1—Cl1	157.69 (4)	C9—C10—H10B	109.5
N4—Ti1—Cl2	102.08 (4)	H10A—C10—H10B	108.1
N1—Ti1—Cl2	92.76 (4)	C10—C11—C12	110.97 (16)
N2—Ti1—Cl2	156.46 (4)	C10—C11—H11A	109.4
N5—Ti1—Cl2	92.70 (4)	C12—C11—H11A	109.4
Cl1—Ti1—Cl2	93.17 (2)	C10—C11—H11B	109.4
N4—Ti1—C1	131.72 (5)	C12—C11—H11B	109.4
N1—Ti1—C1	32.13 (5)	H11A—C11—H11B	108.0
N2—Ti1—C1	31.98 (5)	N3—C12—C11	109.07 (15)
N5—Ti1—C1	99.88 (5)	N3—C12—H12A	109.9
Cl1—Ti1—C1	94.34 (4)	C11—C12—H12A	109.9
Cl2—Ti1—C1	124.85 (4)	N3—C12—H12B	109.9
N4—Ti1—C13	32.50 (5)	C11—C12—H12B	109.9
N1—Ti1—C13	131.67 (6)	H12A—C12—H12B	108.3
N2—Ti1—C13	94.39 (5)	N5—C13—N4	109.59 (14)
N5—Ti1—C13	31.56 (5)	N5—C13—N6	128.95 (14)
Cl1—Ti1—C13	126.14 (4)	N4—C13—N6	121.46 (14)
Cl2—Ti1—C13	99.95 (4)	N5—C13—Ti1	57.31 (8)
C1—Ti1—C13	118.19 (5)	N4—C13—Ti1	52.39 (8)
C1—N1—C2	123.07 (14)	N6—C13—Ti1	173.14 (12)
C1—N1—Ti1	92.95 (10)	N4—C14—C16	111.48 (14)
C2—N1—Ti1	143.91 (11)	N4—C14—C15	109.93 (14)
C1—N2—C5	123.23 (14)	C16—C14—C15	111.42 (15)
C1—N2—Ti1	92.28 (10)	N4—C14—H14	108.0
C5—N2—Ti1	137.87 (12)	C16—C14—H14	108.0
C1—N3—C12	121.71 (14)	C15—C14—H14	108.0
C1—N3—C8	121.35 (14)	C14—C15—H15A	109.5
C12—N3—C8	113.86 (14)	C14—C15—H15B	109.5
C13—N4—C14	121.05 (13)	H15A—C15—H15B	109.5
C13—N4—Ti1	95.10 (10)	C14—C15—H15C	109.5
C14—N4—Ti1	137.93 (10)	H15A—C15—H15C	109.5
C13—N5—C17	124.45 (14)	H15B—C15—H15C	109.5
C13—N5—Ti1	91.13 (10)	C14—C16—H16A	109.5
C17—N5—Ti1	141.22 (11)	C14—C16—H16B	109.5
C13—N6—C24	124.54 (14)	H16A—C16—H16B	109.5
C13—N6—C20	118.69 (13)	C14—C16—H16C	109.5
C24—N6—C20	113.94 (14)	H16A—C16—H16C	109.5
N2—C1—N1	110.09 (14)	H16B—C16—H16C	109.5
N2—C1—N3	126.28 (15)	N5—C17—C19	111.43 (15)
N1—C1—N3	123.62 (15)	N5—C17—C18	108.81 (15)
N2—C1—Ti1	55.74 (8)	C19—C17—C18	111.69 (17)
N1—C1—Ti1	54.92 (8)	N5—C17—H17	108.3
N3—C1—Ti1	173.93 (12)	C19—C17—H17	108.3
N1—C2—C3	110.11 (15)	C18—C17—H17	108.3
N1—C2—C4	111.19 (15)	C17—C18—H18A	109.5
C3—C2—C4	110.72 (17)	C17—C18—H18B	109.5
N1—C2—H2	108.2	H18A—C18—H18B	109.5
C3—C2—H2	108.2	C17—C18—H18C	109.5
C4—C2—H2	108.2	H18A—C18—H18C	109.5
C2—C3—H3A	109.5	H18B—C18—H18C	109.5
C2—C3—H3B	109.5	C17—C19—H19A	109.5
H3A—C3—H3B	109.5	C17—C19—H19B	109.5
C2—C3—H3C	109.5	H19A—C19—H19B	109.5
H3A—C3—H3C	109.5	C17—C19—H19C	109.5
H3B—C3—H3C	109.5	H19A—C19—H19C	109.5
C2—C4—H4A	109.5	H19B—C19—H19C	109.5
C2—C4—H4B	109.5	N6—C20—C21	111.62 (14)
H4A—C4—H4B	109.5	N6—C20—H20A	109.3
C2—C4—H4C	109.5	C21—C20—H20A	109.3
H4A—C4—H4C	109.5	N6—C20—H20B	109.3
H4B—C4—H4C	109.5	C21—C20—H20B	109.3
N2—C5—C7	110.00 (16)	H20A—C20—H20B	108.0
N2—C5—C6	112.07 (16)	C22—C21—C20	110.55 (16)
C7—C5—C6	113.2 (2)	C22—C21—H21A	109.5
N2—C5—H5	107.1	C20—C21—H21A	109.5
C7—C5—H5	107.1	C22—C21—H21B	109.5
C6—C5—H5	107.1	C20—C21—H21B	109.5
C5—C6—H6A	109.5	H21A—C21—H21B	108.1
C5—C6—H6B	109.5	C21—C22—C23	110.28 (15)
H6A—C6—H6B	109.5	C21—C22—H22A	109.6
C5—C6—H6C	109.5	C23—C22—H22A	109.6
H6A—C6—H6C	109.5	C21—C22—H22B	109.6
H6B—C6—H6C	109.5	C23—C22—H22B	109.6
C5—C7—H7A	109.5	H22A—C22—H22B	108.1
C5—C7—H7B	109.5	C22—C23—C24	111.17 (17)
H7A—C7—H7B	109.5	C22—C23—H23A	109.4
C5—C7—H7C	109.5	C24—C23—H23A	109.4
H7A—C7—H7C	109.5	C22—C23—H23B	109.4
H7B—C7—H7C	109.5	C24—C23—H23B	109.4
N3—C8—C9	111.14 (15)	H23A—C23—H23B	108.0
N3—C8—H8A	109.4	N6—C24—C23	109.52 (15)
C9—C8—H8A	109.4	N6—C24—H24A	109.8
N3—C8—H8B	109.4	C23—C24—H24A	109.8
C9—C8—H8B	109.4	N6—C24—H24B	109.8
H8A—C8—H8B	108.0	C23—C24—H24B	109.8
C8—C9—C10	110.60 (16)	H24A—C24—H24B	108.2
			
N4—Ti1—N1—C1	45.1 (2)	Ti1—N1—C1—N2	−8.40 (14)
N2—Ti1—N1—C1	5.62 (9)	C2—N1—C1—N3	−4.8 (3)
N5—Ti1—N1—C1	89.37 (10)	Ti1—N1—C1—N3	172.86 (14)
Cl1—Ti1—N1—C1	−83.61 (10)	C8—N3—C1—N2	120.40 (19)
Cl2—Ti1—N1—C1	−177.27 (9)	C12—N3—C1—N1	139.97 (18)
N4—Ti1—N1—C2	−138.13 (19)	C8—N3—C1—N1	−61.1 (2)
N2—Ti1—N1—C2	−177.7 (2)	C1—N1—C2—C3	114.12 (18)
N5—Ti1—N1—C2	−93.9 (2)	Ti1—N1—C2—C3	−62.0 (3)
Cl1—Ti1—N1—C2	93.1 (2)	C1—N1—C2—C4	−122.78 (19)
Cl2—Ti1—N1—C2	−0.5 (2)	Ti1—N1—C2—C4	61.1 (3)
C1—Ti1—N1—C2	176.7 (3)	C1—N2—C5—C7	166.3 (2)
N4—Ti1—N2—C1	−172.76 (10)	Ti1—N2—C5—C7	−51.1 (3)
N1—Ti1—N2—C1	−5.64 (9)	C1—N2—C5—C6	−66.9 (2)
N5—Ti1—N2—C1	−109.22 (10)	Ti1—N2—C5—C6	75.8 (2)
Cl1—Ti1—N2—C1	92.88 (9)	C1—N3—C8—C9	141.66 (17)
Cl2—Ti1—N2—C1	−12.88 (17)	C12—N3—C8—C9	−57.8 (2)
N4—Ti1—N2—C5	37.75 (18)	N3—C8—C9—C10	53.3 (2)
N1—Ti1—N2—C5	−155.13 (19)	C8—C9—C10—C11	−52.9 (2)
N5—Ti1—N2—C5	101.29 (17)	C9—C10—C11—C12	55.2 (2)
Cl1—Ti1—N2—C5	−56.61 (17)	C1—N3—C12—C11	−140.78 (17)
Cl2—Ti1—N2—C5	−162.37 (13)	C8—N3—C12—C11	58.8 (2)
C1—Ti1—N2—C5	−149.5 (2)	C10—C11—C12—N3	−56.7 (2)
N1—Ti1—N4—C13	46.9 (2)	C17—N5—C13—N4	−167.17 (15)
N2—Ti1—N4—C13	82.33 (10)	Ti1—N5—C13—N4	−3.61 (12)
N5—Ti1—N4—C13	−2.50 (9)	C17—N5—C13—N6	13.0 (3)
Cl1—Ti1—N4—C13	176.36 (9)	Ti1—N5—C13—N6	176.54 (15)
Cl2—Ti1—N4—C13	−89.59 (9)	C14—N4—C13—N5	−153.63 (14)
C1—Ti1—N4—C13	77.19 (11)	Ti1—N4—C13—N5	3.84 (13)
N1—Ti1—N4—C14	−162.48 (16)	C14—N4—C13—N6	26.2 (2)
N2—Ti1—N4—C14	−127.01 (16)	Ti1—N4—C13—N6	−176.30 (13)
N5—Ti1—N4—C14	148.17 (18)	C14—N4—C13—Ti1	−157.47 (16)
Cl1—Ti1—N4—C14	−32.97 (16)	C24—N6—C13—N5	31.7 (3)
Cl2—Ti1—N4—C14	61.07 (16)	C20—N6—C13—N5	−128.08 (18)
C1—Ti1—N4—C14	−132.14 (15)	C24—N6—C13—N4	−148.16 (16)
N4—Ti1—N5—C13	2.57 (9)	C20—N6—C13—N4	52.1 (2)
N1—Ti1—N5—C13	−161.90 (9)	C13—N4—C14—C16	−132.18 (16)
N2—Ti1—N5—C13	−98.73 (10)	Ti1—N4—C14—C16	82.54 (19)
Cl1—Ti1—N5—C13	−0.42 (17)	C13—N4—C14—C15	103.75 (17)
Cl2—Ti1—N5—C13	104.69 (9)	Ti1—N4—C14—C15	−41.5 (2)
C1—Ti1—N5—C13	−129.24 (10)	C13—N5—C17—C19	118.58 (18)
N4—Ti1—N5—C17	160.7 (2)	Ti1—N5—C17—C19	−34.6 (3)
N1—Ti1—N5—C17	−3.78 (19)	C13—N5—C17—C18	−117.83 (18)
N2—Ti1—N5—C17	59.40 (18)	Ti1—N5—C17—C18	89.0 (2)
Cl1—Ti1—N5—C17	157.70 (14)	C13—N6—C20—C21	−142.13 (15)
Cl2—Ti1—N5—C17	−97.19 (18)	C24—N6—C20—C21	56.0 (2)
C13—Ti1—N5—C17	158.1 (2)	N6—C20—C21—C22	−53.4 (2)
C5—N2—C1—N1	164.29 (15)	C20—C21—C22—C23	54.0 (2)
Ti1—N2—C1—N1	8.32 (14)	C21—C22—C23—C24	−56.3 (2)
C5—N2—C1—N3	−17.0 (3)	C13—N6—C24—C23	142.54 (17)
Ti1—N2—C1—N3	−172.98 (15)	C20—N6—C24—C23	−56.9 (2)
C2—N1—C1—N2	173.91 (15)	C22—C23—C24—N6	56.7 (2)
